# Biomarcadores séricos para la evaluación de la fibrosis hepática

**DOI:** 10.1515/almed-2023-0172

**Published:** 2024-02-14

**Authors:** Julia Maroto-García, Ana Moreno-Álvarez, María P. Sanz de Pedro, Antonio Buño-Soto, Álvaro González

**Affiliations:** Departamento de Bioquímica, Clínica Universidad de Navarra, Pamplona, España; Departamento de Análisis Clínicos, Hospital Universitario La Paz, Madrid, España; Instituto de investigación en salud del Hospital La (IdiPaz), Madrid, España; Instituto Navarro de investigación en salud (IdiSNA), Pamplona, España

**Keywords:** fibrosis hepática, biomarcadores de fibrosis hepática, biomarcadores no invasivos, biomarcadores séricos

## Abstract

La fibrosis hepática se desarrolla como respuesta a la presencia de daño hepático crónico de diferentes etiologías, provocando un desequilibrio entre la síntesis y degeneración de la matriz extracelular y la desregulación de diversos mecanismos fisiológicos. En los estadios iniciales de las patologías crónicas, el hígado posee una elevada capacidad de regeneración, por lo que la detección temprana de la fibrosis hepática resulta esencial. En este contexto, es preciso contar con herramientas sencillas y económicas que permitan detectar la fibrosis hepática en sus fases iniciales. Para evaluar la fibrosis hepática, se han propuesto multitud de biomarcadores séricos no invasivos, tanto directos, como el ácido hialurónico o las metaloproteasas, como indirectos. Así mismo, se han desarrollado diversas fórmulas que combinan dichos biomarcadores junto con parámetros demográficos, como el índice FIB-4, el índice de fibrosis en la enfermedad de hígado graso no alcohólico (NFS, por sus siglas en inglés), la prueba ELF o el score de fibrosis Hepamet (HFS, por sus siglas en inglés). En el presente manuscrito, realizamos una revisión crítica del valor diagnóstico y pronóstico de los diferentes biomarcadores séricos y fórmulas actualmente existentes.

## Introducción

La fibrosis hepática se desarrolla como consecuencia de la presencia de daño hepático crónico de diversas etiologías, entre las que se encuentran la hepatitis vírica, el abuso de alcohol, las enfermedades metabólicas como la enfermedad del hígado graso no alcohólico (NAFLD, de sus siglas en inglés), actualmente llamada enfermedad hepática esteatósica asociada a disfunción metabólica (MASLD, de sus siglas en inglés) [1], las enfermedades autoinmunes y las enfermedades hepáticas colestásicas [2, 3]. Esta patología se desarrolla debido a la desregulación del mecanismo fisiológico de remodelado, la activación de los miofibroblastos, y la formación de una cicatriz fibrótica que, con el tiempo, puede acabar produciendo cirrosis [4]. En todas las patologías de fibrosis hepática, se produce un desequilibrio entre la síntesis y la degeneración de la matriz extracelular (MEC), que afecta a la estructura y propiedades del hígado [5]. Aunque el hígado tiene una gran capacidad de regeneración, cuando el daño es persistente, dicha regeneración evoluciona hacia enfermedades crónicas como la fibrosis, que se caracteriza por la acumulación excesiva de MEC [6, 7]. La fibrosis hepática puede ser reversible, especialmente cuando se encuentra en sus fases iniciales [4], antes de que se desarrolle cirrosis y se produzca un fallo orgánico. De este modo, resulta crucial establecer un tratamiento adecuado lo antes posible. Tal como se demuestra en los estudios tanto en modelos experimentales como en pacientes, la regeneración del hígado se ve limitada en la enfermedad hepática avanzada [8].

**Figura 1:**
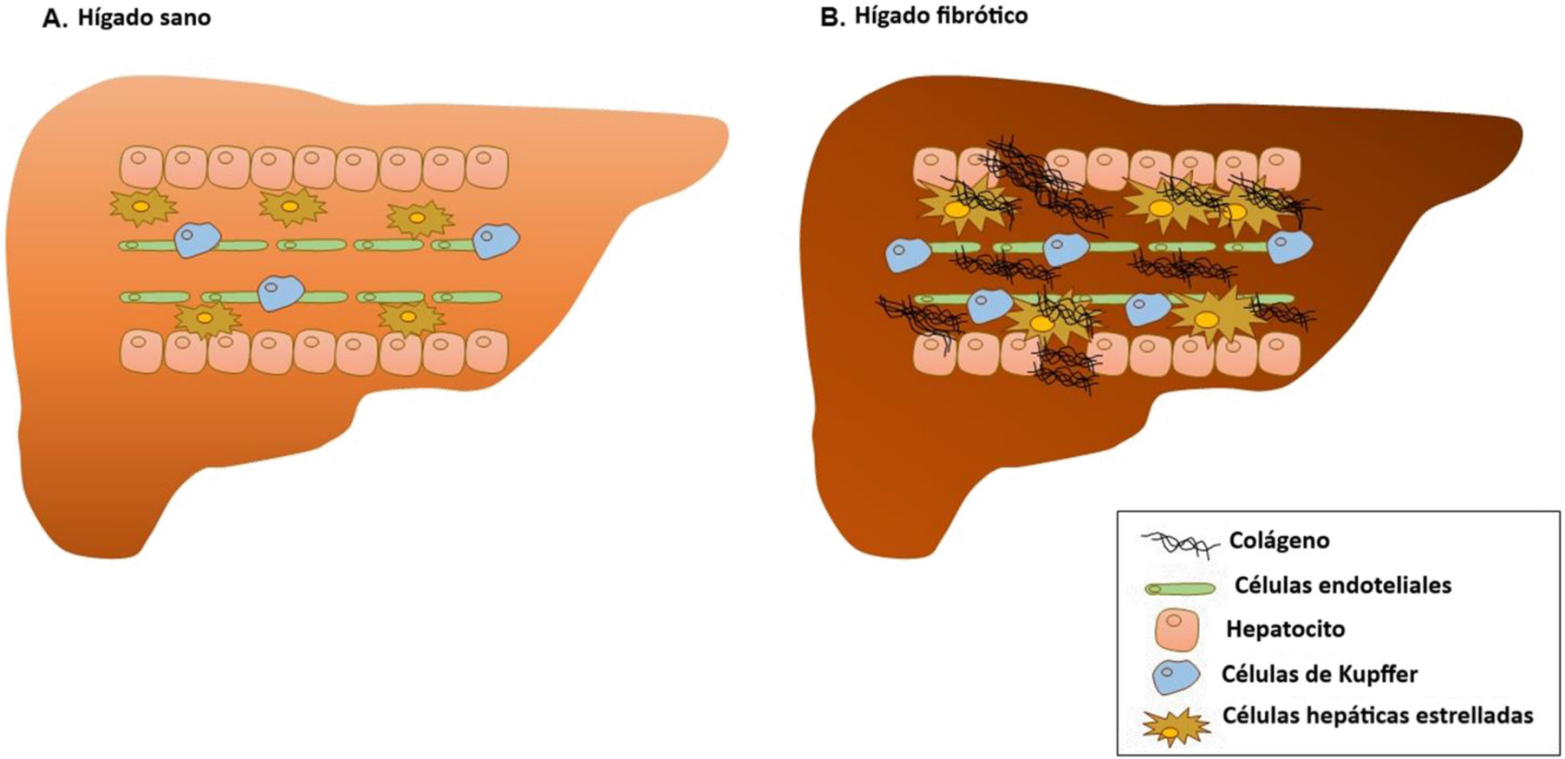
Diferencias entre un hígado sano y un hígado fibrótico.

El daño hepático produce una lesión a nivel de los hepatocitos y afecta a la homeostasis, provocando inflamación [9]. Esta situación provoca una respuesta proinflamatoria de las células de Kupffer, así como la infiltración de las células inmunes, que favorecen la activación y diferenciación de las células estrelladas hepáticas (CEH) en miofibroblastos productores de colágeno [10, 11]. Las CEH controlan la remodelación de la MEC, un proceso que suelen equilibrar los mecanismos antifibróticos, que desactivan el miofibroblasto o estimulan su apóptosis [10]. En las enfermedades hepáticas crónicas, la activación del miofibroblasto provoca una reducción de las metaloproteinasas (MMP, por sus siglas en inglés), la elevación de los inhibidores de MMP (TIMP), y la secreción de la proteína de unión a Mac-2 con aglutinina positiva de Wisteria floribunda (WFA+-M2BP) [12], que están implicadas en la degradación de la MEC. Las MMP son las principales enzimas implicadas en la degradación de la MEC, teniendo las TIMP capacidad para regular las actividades proteolíticas de las MMP en los tejidos [13]. Así mismo, las CEH activadas son los factores que más contribuyen a la acumulación de colágeno en el espacio de Disse, provocando el engrosamiento gradual de dicho espacio, y por tanto un incremento de la presión portal. Como consecuencia, se produce una acumulación excesiva de colágeno, que afecta a la capacidad de regeneración de la matriz, derivando en un incremento de la rigidez hepática [14] (Figura 1).

Actualmente, el método de referencia para evaluar el grado de fibrosis hepática sigue siendo la biopsia hepática, en la que se aplican diversos sistemas de puntuación histopatológica, como METAVIR, que es la más utilizada, en la que se establecen cuatro fases de evolución de la fibrosis hepática: F0, sin fibrosis; F1, fibrosis leve (fibrosis portal sin septos); F2, fibrosis moderada (fibrosis portal con escasos septos); F3, fibrosis avanzada (numerosos septos sin cirrosis); y F4, cirrosis [15]. Las limitaciones de la biopsia hepática son ampliamente conocidas: invasividad de la técnica, mala tolerancia, variabilidad en la toma de muestras, coste elevado, y variabilidad interobservador en la interpretación [16]. En este contexto, es necesario desarrollar e incluir otros biomarcadores no invasivos para el diagnóstico y evaluación de la evolución de la enfermedad hepática.

En este manuscrito hemos llevado a cabo una revisión crítica de los biomarcadores séricos de fibrosis hepática. Los biomarcadores directos son aquellos relacionados con el proceso de formación y degradación de la MEC o con la patogénesis molecular de la fibrogénesis y la fibrinólisis. Por otra parte, los biomarcadores indirectos se definen como aquellos parámetros bioquímicos que reflejan alteraciones en la función hepática y daño hepático [17].

## Biomarcadores indirectos

### Enzimas hepáticas

La alanina aminotransferasa (ALT) y la aspartato aminotransferasa (AST) proporcionan información sobre el daño al hepatocito. Los pacientes con enfermedad hepática avanzada presentan niveles reducidos de ALT [18], asociados a una elevación de AST y de la ratio AST/ALT [19]. Además, hasta el 80 % de los pacientes con MASLD presentan concentraciones normales de aminotransferasa, por lo que este parámetro no se considera fiable como factor predictivo de enfermedad hepática avanzada [20]. Existe evidencia de que los pacientes con alteraciones en el metabolismo de la glucosa y resistencia a la insulina y con niveles normales de ALT, podrían desarrollar MASLD. Del mismo modo, los niveles normales de aminotransferasas no deben excluir la realización de estudios complementarios, como pruebas de imagen o una biopsia hepática [21]. La ratio AST/ALT se ha utilizado tradicionalmente para determinar el riesgo de cirrosis en pacientes con hepatitis vírica crónica [22, 23], esteatothepatitis no alcohólica (NASH), enfermedad hepática alcohólica (ALD) [24, 25] y cirrosis biliar primaria (CBP) [26]. Sin embargo, actualmente, las diferentes fases de la fibrosis no se pueden identificar meramente con el uso de este cociente, ya que no distingue entre fibrosis moderada y grave [27, 28].

### Índice APRI

En el pasado, se solía calcular el la ratio AST/plaquetas (índice APRI) [25], [26], [27], [28], [29] para evaluar si los pacientes con VHC presentaban fibrosis hepática o no, así como para diferenciar las distintas fases de fibrosis hepática de la cirrosis [30, 31]. Recientemente, se ha propuesto un índice APRI modificado (m-APRI), que incorpora la edad y los niveles de albúmina sérica a la fórmula APRI (Tabla 1) [32]. Se ha demostrado que este m-APRI mejora la predicción de fibrosis avanzada y cirrosis en la hepatitis vírica [33].

**Tabla 1:** *Scores* utilizados en la evaluación y estadificación de la fibrosis hepática.

Score/índice	Fórmula
APRI	AST (UI/L)/valor AST en el límite superior de normalidad (UI/L)/recuento de plaquetas (10^9^/L)×100
m-APRI	(Edad (años)×(AST (UI/L)/valor AST en el límite superior de normalidad(UI/L))/ (Albúmina × recuento de plaquetas(10^9^/L))×100
BARD score	AST/ALT>0,8 2 puntos
	IMC>28 1 punto
	Diagnóstico de diabetes 1 punto
Índice forns	7,811−(3,131×ln(recuento de plaquetas(10^9^/L)))+(0,781×In(GGT (UI/L)))+(3,467×ln(Edad (años)))−(0,014×(colesterol (mg/dL)))
FIB-4	AST (UI/L)×Edad (años)/(recuento de plaquetas(10^9^/L)×√(ALT (UI/L))
NFS	−1,675+(0,037×Edad (años))+(0,094×IMC (kg/m^2^))+(1,13×GAA/diabetes (sí=1, no=2))+(0,99×ratio AST/ALT)−(0,013×plaquetas (10^9^/L))−(0,66×albúmina (g/dL))
FibroTest	(4,467×log (α2-MG))−(1,357×log (haptoglobina))+(1,017×log (GGT))+(00,281×Edad (años))+(1,737×log (bilirrubina total))−(1,184×apoA1)+(0,301×sexo (masculino=1, femenino=0))−5,540
Fibrometer NAFLD	(04,184×glucosa (mmol/L))+(00,701×AST (UI/L))+(00,008×ferritina (μg/L))−(00,102×plaquetas (10^9^/L))−(00,260×ALT (UI/L))+(00,459×peso corporal (kg)+00,842 Edad (años))+11,6226
Hepascore	Y/(1+Y)
	Donde Y=exp (−4,185818−(00,249×Edad)+(0,7464×sexo)+(10,039×α2-MG)+(00,302×HA)+(00,691×bilirrubina total)−(00,012×GGT))
HFS	1/(1+e^[5,390−0,986×Edad [45–64 años]−1,719×Edad [≥65 años]+0,875×Sexo masculino−0,896×AST [35–69 UI/L]−2,126×AST [≥70 UI/L]−0,027×Albúmina [4–4,49 g/dL]−0,897×Albúmina [<4 g/dL]−0,899×HOMA−R [2−3,99 sin Diabetes Mellitus]−1,497×HOMA−R [≥4 sin Diabetes Mellitus]−2,184×Diabetes Mellitus−0,882×plaquetas×1,000/μL [155−219]−2,233×plaquetas×1,000/μL [<155]^),
Índice Benlloch	1/1+e^−12,698+(0,097×(ratio Albúmina/proteínas totales))−(1,356×(tiempo de protrombina))−(0,004×(AST))−(0,02×(tiempo desde trasplante hepático))^
ADAPT score	exp (log_10_ ((Edad×PRO-C3)/√Recuento de plaquetas)+diabetes (0=ausente; 1=presente)
ELF	2,494+0,846 ln(HA)+0,735 ln(PIIINP)+0,391 ln(TIMP-1)
Modelo CHI3L1	0,032×AST−0,012×plaquetas+0,012×HA+0,846×log10 (CHI3L1)−4,752
M2BPGi COI	([M2BPGi]_muestra_−[M2BPGi]_control negativo_)/([M2BPGi]_control positivo_−[M2BPGi]_control negativo_)

α2-MG, alfa 2 macroglobulina; ALT, alanina aminotransferasa; ApoA1, apolipoproteína A1; APRI, índice de relación AST/plaquetas; AST, aspartato aminotransferasa, IMC, índice de masa corporal; CHI3L1, proteína 1 similar a quitinasa 3; ELF, enhanced liver fibrosis; GGT, gamma-glutamiltranspeptidasa; AH, ácido hialurónico; HFS, *score* de fibrosis de Hepamet; HOMA-R, evaluación del modelo homeostático de resistencia a la insulina; m-APRI, APRI modificado; M2BPGi, isómero de glicosilación de la proteína de unión a Mac-2; M2BPGi COI, índice de corte del isómero de glicosilación de la proteína de unión Mac-2; NFS, *score* de fibrosis hepática de la enfermedad del hígado graso no alcohólico; GAA, glucosa alterada en ayunas; PIIINP, propéptido del procolágeno tipo III; TIMP-1, inhibidor de metaloproteinasas de matriz tipo 1.

### BARD score

Este *score* fue propuesto por Harrison y col. e incluye la presencia de diabetes mellitus tipo 2, el índice de masa corporal (IMC) del paciente y la actividad de las enzimas hepáticas en suero, determinada a través de la relación AST/ALT [34]. En los pacientes con MASLD, BARD tiene un valor predictivo negativo (VPM) elevado del 96 % [34]. Recientemente, Park y col. [35], han descrito en pacientes con MASLD la asociación entre fibrosis hepática avanzada, evaluada mediante la puntuación BARD, y un mayor riesgo de enfermedad cardiovascular y mortalidad, sugiriendo su relación con la inflamación miocárdica y el ictus isquémico.

### Forns index

El índice de Forns es un sistema de puntuación que combina la edad, la gamma-glutamil transferasa (GGT), el colesterol y el recuento de plaquetas, cuya utilidad ha sido demostrada en la identificación de pacientes con hepatitis C crónica sin fibrosis hepática moderada [36]. Así mismo, el índice Forns ha sido validado en pacientes con MASLD confirmada mediante biopsia, en poblaciones de pacientes con enfermedad hepática crónica sin cirrosis descompensada o carcinoma hepatocelular [37]. Romero y col [38], obtuvieron una precisión del 95,2 % con el índice Forns a la hora de predecir fibrosis moderada, y del 91,7 % en la detección de fibrosis avanzada en pacientes con el genotipo 1 de hepatitis C crónica (CHC). De este modo, existe evidencia de que el índice Forns es un factor predictivo de morbilidades y mortalidad en pacientes con MASDL, similar a APRI [39].

### FIB-4

FIB-4 es un índice basado en la edad, la actividad de AST y ALT en suero y la concentración de plaquetas (Tabla 1). Este es probablemente el índice sérico más ampliamente utilizado en el cribado inicial de la fibrosis hepática, estando recomendado su uso en las guías de práctica clínica para la MASLD, como la Guía de Práctica sobre la evaluación clínica y manejo de la enfermedad de hígado graso no alcoholico de la American Association for the Study of Liver Diseases (AASLD) [1, 40]. El punto de corte más aceptado para FIB-4 para detectar fibrosis avanzada es 2,67 [41], aunque algunos estudios lo incrementan hasta 3,25 [42] (Tabla 2). Itakura y col [43] establecieron la tasa de precisión del FIB-4 en 71 % para el diagnóstico de cirrosis por infección por el VHB, y 75 % en pacientes con infección por el VHC. Aunque en diversos meta-análisis también se ha concluido que FIB-4, así como APRI, fueron moderadamente efectivos para la evaluación de la fase de fibrosis en la hepatitis B crónica [44], [45], [46], FIB-4 mostró mayor precisión diagnóstica que APRI, a la hora de predecir fibrosis moderada o avanzada o un diagnóstico de cirrosis [46]. Tal como demuestran distintos estudios, FIB-4 posee un elevado valor diagnóstico a la hora de evaluar la cirrosis y la fibrosis moderada o grave [47], [48], [49], [50], [51], [52], [53], [54]. FIB-4 es una herramienta útil en el cribado de la fibrosis hepática, debido a su viabilidad y elevado VPN [55]. Así mismo, se ha propuesto un punto de corte de 1,3 para descartar la fibrosis hepática avanzada [56]. Por otro lado, su especifidad para la fibrosis avanzada es menor en pacientes ≥65 años, derivando en una mayor tasa de falsos positivos, por lo que el punto de corte para el FIB-4 en este grupo de edad se ha elevado a 2 [57]. Aunque la utilidad de este biomarcador para descartar fibrosis hepática avanzada en pacientes con patologías de gran prevalencia, como la diabetes o la MASLD no se puede descartar únicamente en función del FIB-4. De este modo, en caso de elevada sospecha, se recomienda la evaluación o empleo de otros métodos más específicos [58, 59].

**Tabla 2:** Puntos de corte de los biomarcadores séricos de fibrosis hepática establecidos en la literatura.

Score/índice	Pacientes/cohorte	Diagnóstico	Predice/descarta	AUC	Punto de corte	Sensibilidad, %	Especifidad, %	VPP, %	VPN, %	Referencias
AST/ALT	MASLD	Fibrosis avanzada	Predice	0,83 (0,74–0,91)	>0,8	74	78	44	93	[60]
APRI	Pacientes con hepatitis vírica crónica	Fibrosis significativa	Predice	0,72 (0,69–0,75)	>0,5	79,9	48,4	67,3	64,4	[61]
	MASLD	Fibrosis avanzada	Predice	0,67 (0,54–0,8)	>1	27	89	37	84	[60]
		Cirrosis	Predice	0,77 (0,73–0,81)	>2	45,2	88,4	38,7	90,9	[61]
mAPRI	MASLD	Fibrosis avanzada	Predice	0,84 (0,78–0,89)	>5,84	77,8	79,8	75,7	81,6	[32]
		Cirrosis	Predice	0,83 (0,74–0,87)	>9	67,3	85,7	67,3	85,7	[32]
BARD	MASLD	Fibrosis avanzada	Predice	0,77 (0,68–0,87)	>2	89	44	27	95	[60]
Índice de forns	ALD	Fibrosis avanzada	Predice	0,83 (0,78–0,89)	>6,9	67	89	55	93	[62]
FIB-4	MASLD (edad<65)	Fibrosis avanzada	Descarta	0,86 (0,78–0,94)	≤1,3	85	65	36	95	[42, 60]
			Predice		>3,25	26	98	75	85	[60]
	MASLD (edad>65)	Fibrosis avanzada	Predice	NI	>2	77	70	12	98	[57]
	Pacientes con coinfección de VIH/VHC	Fibrosis avanzada	Descarta	0,737	<1,45	66,7	71,2	38	89	[47]
			Predice		>3,25	26	96,6	64,5	82,6	[47]
		Fibrosis avanzada	Descarta	0,802 (0,758–0,847)	<1,3	74	71	43	90	[41]
			Predice		>2,67	33	98	80	83	[41]
NSF	MASLD	Fibrosis avanzada	Descarta	0,81 (0,71–0,91)	<-1,455	78	58	30	92	[60]
			Predice		>0,676	33	98	79	86	[60]
		Fibrosis avanzada	Descarta	0,84 (0,81–0,88)	<-1,455	77	71	52	88	[63]
			Predice		>0,676	43	96	82	80	[63]
Fibrotest	Pacientes con hepatitis vírica crónica	Fibrosis significativa	Predice	0,78 (0,75–0,81)	>0,48	67,4	75,3	78,7	63,1	[61]
	Pacientes con hepatitis vírica crónica	Cirrosis	Predice	0,82 (0,79–0,85)	>0,74	62,6	84,4	40,1	93,1	[61]
Gp73	Pacientes con VHB crónica	Significant liver inflammation	Predice	0,806 (0,748–0,856)	>85,7 ng/mL	43,59	97,18	89,5	75,8	[64]
	Pacientes con VHB crónica	Fibrosis significativa	Predice	0,742 (0,679–0,799)	>84,49 ng/mL	30,70	96,23	89,74	56,35	[64]
HFS	MASLD	Fibrosis avanzada	Descarta	NI	<0,12	74,6	75,5	49,8	90,1	[65]
			Predice	NI	≥0,47	34,6	96,7	77,2	81,9	[65]
Índice DE Benlloch	Pacientes trasplantados con VHC crónica	Fibrosis significativa	Descarta	0,84	≤0,2	87	71	49	95	[66]
		Fibrosis significativa	Predice	0,84	≥0,8	17	99	80	79	[66]
HA	Patologías hepáticas crónicas	Fibrosis avanzada	Predice	NI	>90 μg/L	80,4	70,2	86,7	59,8	[67]
	Patologías hepáticas crónicas	Cirrosis	Predice	NI	>210 μg/L	96,2	85,3	65,4	98,8	[67]
PCIIINP	Patologías hepáticas crónicas	Fibrosis avanzada	Predice	NI	>90 μg/L	82	60,8	83,5	58,4	[67]
	Patologías hepáticas crónicas	Cirrosis	Predice	NI	>150 μg/L	76,4	68,7	40,4	91,3	[67]
CIV	Patologías hepáticas crónicas	Fibrosis avanzada	Predice	NI	>75 μg/L	63,1	83,8	90,4	48,4	[67]
	Patologías hepáticas crónicas	Cirrosis	Predice	NI	>90 μg/L	80	75,8	47,8	93,2	[67]
PRO-c3	ALD	Fibrosis avanzada	Predice	0,85 (0,79–0,90)	>15,6	81	73	38	95	[62]
	Pacientes con VHC crónica	Fibrosis avanzada	Predice	0,72 (0,65–0,78)	>20,2	71,4	71,9	NI	NI	[68]
ADAPT score	ALD	Fibrosis avanzada	Predice	0,88 (0,83–0,93)	>6,328	86	78	44	97	[62]
	MASLD	Fibrosis avanzada	Predice	0,86 (0,79–0,91)	>6,328	NI	NI	48,4	96,6	[69]
CHI3L1 o YKL-40	MASLD	Fibrosis avanzada	Predice	0,764	>165 μg/L	70	76,8	NI	NI	[70]
	ALD	Fibrosis avanzada	Predice	NI	>330 μg/L	88,5	50,8	NI	NI	[71]
	VHB	Fibrosis avanzada	Predice	0,97	>68,75 μg/L	95,2	89,7	NI	NI	[72]
	VHC	Fibrosis avanzada	Predice	0,809	>186,4 μg/L	78	81	NI	NI	[73]
M2BPGi COI	VHB	Fibrosis significativa	Predice	0,653 (0,608–0,698)	>0,25	74,8	47,3	NI	NI	[74]
	VHB	Fibrosis avanzada	Predice	0,59 (0,50–0,67)	≥3,0	18,8	98,5	NI	NI	[75]
			Predice	0,795 (0,743–0,848)	>0,45	69,6	74,1	NI	NI	[74]
	VHB	Cirrosis	Predice	0,914 (0,815–1)	>0,96	83,3	92,7	NI	NI	[74]
ELF	Patologías hepáticas crónicas	Severe fibrosis	Predice	0,86 (0,83 - 0,89)	≥10,48	62	89	73	83	[76]
	Fibrosis hepática (cohorte EUROGOLF)	Fibrosis leve	Predice	NI	>7,7	85	38	NI	NI	[77]
	Fibrosis hepática (cohorte EUROGOLF)	Fibrosis avanzada	Predice	NI	>9,8	65	90	NI	NI	[77]
	Fibrosis hepática (cohorte EUROGOLF)	Cirrosis	Predice	NI	≥11,3	38	97	NI	NI	[77]
	VHB	Advance fibrosis	Predice	NI	>9,8	62	66	55	72	[78]
Hepascore	VHC	Fibrosis significativa	Predice	0,81	≥0,55	82	65	70	78	[79]
	Pacientes con hepatitis vírica crónica	Fibrosis significativa	Predice	0,78 (0,75–0,80)	>0,5	52,9	86,3	83,7	57,9	[61]
	VHC	Fibrosis significativa	Predice	0,852 (0,778–0,926	>0,5	67	92	NI	NI	[80]
	VHC	Fibrosis avanzada	Predice	0,957 (0,918–0,995)	>0,5	95	81	NI	NI	[80]
	VHC	Cirrosis	Predice	0,938 (0,872–1,000	>0,84	71	89	NI	NI	[80]
	Pacientes con hepatitis vírica crónica	Cirrosis	Predice	0,86 (0,83–0,88)	>0,84	59	87,4	43,2	92,9	[61]
Fibrometers	Pacientes con hepatitis vírica crónica	Fibrosis significativa	Predice	0,79 (0,76–0,81)	>0,411	83,1	57,1	72	71,8	[61]
	Pacientes con hepatitis vírica crónica	Cirrosis	Predice	0,86 (0,83–0,89)	>0,442	43,9	95	58,1	91,5	[61]

ALT, alanina aminotransferasa; APRI, índice de relación AST/plaquetas; AST, aspartato aminotransferasa; AUC, área bajo la curva; CHI3L1, proteína 1 similar a quitinasa 3; ELF, Enhanced Liver Fibrosis; Gp73, proteína 73 de Golgi; HA, ácido hialurónico; HFS, *score* de fibrosis Hepamet; VHB, virus de la hepatitis B; VHC, virus de la hepatitis C; M2BPGi COI, punto de corte del isómero de glicosilación de la proteína de unión Mac-2; NAFLD, enfermedad del hígado graso no alcohólico; NFS, *score* de fibrosis NAFLD; VPN, valor predictivo negativo; NI, no informado; OELF, ELF original; PIIINP, propéptido N-terminal de procoledadn tipo III; VPP, valor predictivo positivo.

### NAFLD *score* de fibrosis

NAFLD *score* considera los mismos parámetros que el FIB-4, añadiendo el IMC, la presencia o no de diabetes y la albúmina (Tabla 1). Su uso viene recomendado para el diagnóstico de fibrosis hepática avanzada, en la guía de práctica clínica de la EASL-EASD-EASO [81] para el manejo de MASLD, ya que este *score* tiene un área bajo la curva (AUROC) >0,8, similar al del FIB-4 (Tabla 2) [60, 63]. Sin embargo, a pesar de su viabilidad, aún se considera que, tanto FIB-4 como NFS, son herramientas subóptimas, ya que presentan una proporción considerable de falsos positivos y falsos negativos cuando se aplican a la población general, por lo que únicamente deberían ser empleados en las poblaciones de riesgo [82].

### Fibrotest

FibroTest™ (Biopredictive Paris) consiste en un panel de marcadores, entre los que se encuentran la α2-macroglobulina sérica, apolipoproteína A1, haptoglobina, bilirrubina total y GGT, ajustadas por edad y sexo [83] (Tabla 1). Fibrotest ha sido validado para la estadificación de la fibrosis hepática en enfermedades hepáticas comunes, como la hepatitis B crónica [84], la enfermedad hepática alcohólica [85], y la MASLD [83]. En un metaanálisis reciente [86], se concluyó que Fibrotest tiene un rendimiento aceptable en la detección de cirrosis (AUC 0,92) en los pacientes con MASLD. Sin embargo, ha mostrado una precisión limitada a la hora de predecir fibrosis moderada y avanzada (AUROC=0,77 para las dos patologías). Degos y col. obtuvieron resultados similares en pacientes con hepatitis vírica crónica [61] (Tabla 2). No obstante, Fibrotest presenta buenos valores predictivos para el diagnóstico de fibrosis hepática en pacientes con MASLD, por lo que se incluye en las guías de práctica clínica de la EASL-EASD-EASO [81], así como para la supervivencia sin mortalidad relacionada con enfermedad hepática, mortalidad relacionada con patologías cardíacas y supervivencia global [87].

### Proteína de Golgi 73 (Gp73)

GP73 es una proteína transmembrana liberada por células dañadas, cuyos niveles séricos se encuentran elevados en los pacientes con patologías hepáticas crónicas [88–90]. Se sabe que GP73 está sobreexpresada en pacientes con cirrosis hepática [90], poseyendo un elevado valor diagnóstico en la cirrosis hepática [91, 92]. Recientemente, en pacientes con cirrosis compensada, se ha observado una relación entre niveles elevados de GP73 y peores resultados clínicos, como la descompensación, el desarrollo de hepatocarcinoma, y mortalidad relacionada con patología hepática [93, 94]. Su uso también ha sido validado en pacientes con MASLD [95], ALD y hepatitis vírica [64, 88, 96], lo que demuestra su utilidad para el diagnóstico de fibrosis y cirrosis avanzada, presentando un valor diagnóstico mayor que los índices FIB-4 y APRI [97].

### *Score* de fibrosis Hepamet (HFS)

HFS es un nuevo *score* recientemente propuesto, validado en una población europea multicéntrica caucásica con MASLD confirmada mediante biopsia, que incluye la edad, el sexo, la diabetes, el modelo homeostático para evaluar la resistencia a la insulina (HOMA-IR), AST, albúmina, y las plaquetas [65]. Según el equipo que lo desarrolló, HFS identifica a los pacientes con fibrosis avanzada con mayor precisión que los índices FIB-4 y NFS. Aunque se trata de un nuevo *score*, diversos estudios han validado sus puntos de corte [98–100] y verificado que HFS es el que muestra mayor precisión diagnóstica y mayor valor predictivo negativo, si se compara con los índices NFS y FIB-4 en pacientes con esteatosis hepática metabólica [101]. Además, HFS es tan fiable como NFS y FIB-4 en la predicción de cirrosis, eventos hepáticos a largo plazo, hepatocarcinoma y mortalidad global, con un mayor rendimiento en la predicción de fibrosis moderada y grave. Esto puede explicarse por la inclusión de la presencia de diabetes en su fórmula o el HOMA-IR en los pacientes no diabéticos [102]. Por otro lado, niveles elevados de HFS (punto de corte 0,12) están relacionados con un mayor riesgo de desarrollar diabetes mellitus 2 e hipertensión arterial en pacientes con MASLD, eventos que ni NFS ni FIB-4 pudieron predecir [103].

### OWLiver

La prueba metabolómica patentada OWLiver^®^ (One Way Liver S.L., Bilbao, España) es un análisis de sangre en ayunas que se emplea para determinar el grado de desarrollo de MASLD, mediante la medición de un panel de biomarcadores séricos de triacilgliceroles combinado con el IMC, que representan la grasa e inflamación en el hígado [104]. Los triacilgliceroles se miden mediante cromatografía líquida de alta resolución combinada con espectrometría de masas (UHPLC-MS), considerando posteriormente todos los resultados en un algoritmo, del que se obtiene una puntuación final OWLiver^®^ [105] Owliver distingue entre un hígado normal y fibrosis hepática MASLD, con un AUROC de 0,90 y una elevada sensibilidad, lo que implica una reducida tasa de falsos negativos [106]. Este *score* también distingue la esteatosis simple de la esteatohepatitis, tal como se muestra en el estudio de validación prospectivo, en el que se había diagnosticado previamente a los pacientes mediante biopsia hepática [104]. Según Bril y col [107], comparadas con la biopsia hepática, las pruebas OWLiver^®^ Care y OWLiver^®^ mostraron un rendimiento subóptimo en pacientes con diabetes mellitus tipo 2. La falta de estudios comparativos entre OWLiver^®^ y otros *score*s de fibrosis hepática no invasivos, sumado a la complejidad de la metodología, dificultan su implementación en la práctica clínica.

### Índice de Benlloch

El índice Benlloch es un modelo creado para evaluar a los pacientes con VHC crónico sometidos a trasplante hepático. El propósito del índice Benlloch es evaluar si es necesario o no iniciar una terapia antiviral, así como realizar un seguimiento estrecho de este grupo de pacientes, con el fin de determinar si está indicado un retrasplante [66]. Este índice se basa en cuatro biomarcadores indirectos, entre los que se incluyen AST, tiempo de protrombina, relación albúmina/proteína total, y tiempo transcurrido desde el trasplante hepático (Tabla 1). Se ha demostrado su eficacia con respecto a la biopsia hepática en pacientes con HVC crónica sometidos a trasplante hepático, habiendo mostrado un poder de discriminación aceptable a la hora de distinguir la fibrosis significativa de la avanzada [66].

## Biomarcadores directos

### Proteínas de la matriz extracelular

#### Ácido hialurónico

El ácido hialurónico (AH) es un componente esencial de la matriz extracelular, con una elevada presencia en el hígado [108]. El AH es secretado por diversos tipos de células. Las células del revestimiento sinovial y las CEH son las responsables de su síntesis en el hígado, mientras que las células endoteliales sinusoidales participan en su degradación [109]. Las concentraciones séricas de AH se encuentran elevadas en las enfermedades hepáticas asociadas a la fibrosis, como ALD [109, 110], MASLD [111–113], VHC [114–116], VHB [108, 117], y coinfección de VIH-VHC [118, 119]. De este modo, se puede emplear como biomarcador no invasivo para evaluar la presencia de fibrosis hepática y realizar un seguimiento de la progresión de la enfermedad [109, 120].

#### Propéptido N-terminal del procolágeno tipo III

En un estudio reciente se ha identificado al propéptido N-terminal del procolágeno tipo III (PCIIINP), uno de los principales componentes del tejido conectivo, como un buen marcador de fibrosis hepática en pacientes con diabetes tipo 2 [121]. Los niveles séricos de PCIIINP se encuentran elevados en los pacientes con ALD o VHC y está correlacionado con el estadio de fibrosis hepática [122–124]. Así mismo, existe evidencia de que los niveles plasmáticos de PCIIINP son superiores a los índices APRI o FIB-4 como marcadores no invasivos para la estadificación de la fibrosis hepática en niños y adolescentes con MASLD confirmada mediante biopsia [125].

#### Laminina y colágeno tipo IV

El colágeno tipo IV (CIV) y la laminina (LN) han sido ampliamente estudiados en pacientes con ALD, hepatitis viral y MASLD [111, 126]. Del mismo modo, los niveles séricos de CIV y LN están correlacionados con la fase fibrótica de la infección por VHC y algunos estudios los identifican como biomarcadores no invasivo precisos de fibrosis hepática e inflamación hepática [127]. Por otro lado, la LN posee un menor valor diagnóstico que el AH y el CIV [128].

Algunos autores proponen emplear conjuntamente el AH, PCIIINP y CIV como biomarcadores para un diagnóstico más preciso de fibrosis hepática en diferentes patologías hepáticas crónicas [67]. Por otro lado, Stefano y col observaron que CIV puede predecir la presencia de fibrosis moderada y avanzada en pacientes con MASLD, al haber mostrado mejor AUROC que LN, AH y PCIIINP [129].

#### Sitio de escisión de la N-proteasadel PIIINP

El sitio de escisión de la N-proteasadel PIIINP (PRO-C3) es un marcador sistémico de la formación de colágeno III y de actividad fibroblástica. Así, está directamente relacionado con el desarrollo de fibrosis hepática. Se ha demostrado que este marcador detecta la fibrosis hepática, el índice de progresión y la respuesta a tratamiento en pacientes con enfermedad hepática crónica [68, 130–132]. Inicialmente, se confirmó que PRO-C3 distingue la fibrosis moderada de la severa en pacientes con CHC, e identifica a los pacientes con CHC con progresión de fibrosis con mayor precisión que la prueba habitual, FibroTest [68]. Así mismo, se ha observado que, en la población con ALD y MASLD, la utilidad de PRO-C3 aumenta cuando se emplea un algoritmo llamado ADAPT *score*, que incluye en su fórmula la edad, la presencia de diabetes y el recuento de plaquetas (Tabla 1) [62, 69]. ADAPT *score* es superior a APRI, FIB-4 y NFS, presentando además la ventaja de poder estratificar la fibrosis y la cirrosis, al contrario que otros biomarcadores no invasivos, que solo proporcionan resultados dicotómicos [69].

#### Metaloproteinasas de la matriz (MMP) e inhibidor tisular de metaloproteinasas (TIMP)

TIMP-1 era la única metaloproteinasa que se podría considerar como un factor predictivo independiente de fibrosis histológica, tal como se demostró en un estudio en pacientes con MASLD [133]. Sin embargo, Livzan y col. señalan a TIMP-2 como un posible marcador no invasivo para el diagnóstico de fibrosis hepática en pacientes con MASLD, al haber mostrado una buena correlación con la gravedad de la fibrosis [134]. Boeker y col. observaron que MMP-2 también se puede emplear para detectar la cirrosis con gran eficacia en pacientes con VHC crónica, con mayor valor diagnóstico que el AH o TIMP-1 [135]. Además, se ha propuesto la relación MMP-2/TIMP-1 como un indicador de respuesta a tratamiento con inferón-γ en pacientes con CHC, habiendo sido descrita una mayor reducción de dicha relación en los pacientes con buena respuesta, frente a los que no respondieron o no recibieron tratamiento [136]. Munsterman y col. describieron mayores niveles de TIMP-1 y TIMP-2 en pacientes con fibrosis grave que en aquellos con fibrosis leve o la ausencia de fibrosis en pacientes con MASLD. Así mismo, los autores observaron que MMP-9 era el único componente de MEC que se correlacionaba con la gravedad de la inflamación [137]. Recientemente, se ha identificado a MMP-7 como un marcador independiente de fibrosis hepática, capaz de mejorar el valor diagnóstico en pacientes con MASLD de edad avanzada, si se combina con la prueba mejorada de fibrosis hepática (Enhanced Liver fibrosis, ELF) [138].

#### Proteína 1 similar a la quitinasa 3 (CHI3L1)

CHI3L1, también llamada YKL-40, es una proteína secretada por los macrófagos, neutrófilos, células musculares lisas vasculares, y células tumorales, entre otras, aunque su expresión en el hígado es mayor que en otros tejidos. CHI3L1 realiza diversas funciones, como promover la degradación de la matriz extracelular y la remodelación de los tejidos [139]. Los niveles de CHI3L1 se han relacionado con el grado de fibrosis hepática en pacientes con MASLD [70], ALD [71], VHB [72], y VHC [73]. Huang y col. demostraron que CHI3L1 es un buen marcador a la hora de identificar fibrosis sustancial (AUC=0,94) y fibrosis avanzada (AUC=0,96). Así mismo, determinaron que CHI3L1 es superior al AH, PCIIINP, LN, y CIV para dicho propósito [140]. Saitou y col [73] observaron que los niveles séricos de CHI3L1 son superiores a otros marcadores no invasivos de fibrosis, como CIV, AH, y PCIIINP, a la hora de distinguir la fibrosis avanzada de la fibrosis leve, con un AUC de 0,809, en pacientes con infección de VHC. Así mismo, los autores observaron que dichos niveles se reducían tras la terapia. Se ha propuesto un modelo de CHI3L1, habiéndose demostrado que es superior a los índices APRI y FIB-4, en la predicción de la fibrosis moderada, en pacientes con VHB con niveles de ALT dos veces por debajo del límite superior del rango de normalidad [141].

#### Isómero de glicosilación de proteínas de unión a Mac‐2 (M2BPGi)

M2BPGi es una glicoproteína producida por las CHE, que funciona como mensajera entre las CEH y las células de Kupffer, promoviendo la fibrogénesis [142]. Así, se ha recomendado su uso, al ser un biomarcador preciso para la estadificación de la fibrosis hepática [143, 144]. Los niveles de M2BPGi se expresan en la literatura como un punto de corte, y su utilidad en la fibrosis hepática se ha validado en diversos estudios en pacientes con diferentes patologías, como VHC [145], VHB [75, 146] (Tabla 2), hepatitis autoinmune [147], NASH [148], MASLD [111, 149], atresia biliar [150], cirrosis biliar primaria [151], colangitis esclerosante primaria [152] y mortalidad en la cirrosis hepática [153]. En un estudio en pacientes con VHB, M2PBGi mostró correlación con el grado de fibrosis (F0–F4) y fue superior al recuento de plaquetas, AH, PCIIINP, TIMP-1, FIB-4, APRI, y ELF *score,* a la hora de establecer el grado de fibrosis moderada [154]. Se obtuvieron resultados similares al comparar la relación AST/ALT, APRI, y FIB-4 en la detección de fibrosis hepática avanzada [74]. Además, estudios recientes describen M2BPGi como biomarcador para el seguimiento de los pacientes con fibrosis hepática [155–159]. A los pacientes con niveles elevados de M2BPGi tras tratamiento antiviral se les debe realizar un seguimiento estrecho para detectar el posible desarrollo de hepatocarcinoma [155, 160]. Además, en un estudio en pacientes con VHC, M2BPGi fue superior a FIB-4, a la hora de distinguir los diferentes grados de fibrosis tras tratamiento con antivirales de acción directa [161].

Aunque la utilidad de estos biomarcadores directos está demostrada, su sensibilidad y especifidad aumentan cuando se usan conjuntamente [124], superando las de los algoritmos o *score*s como ELF, Hepascore o Fibrometer, descritos a continuación.

### Fórmulas O índices calculados empleando proteínas de la matriz extracelular

#### Enhanced liver fibrosis (ELF)

ELF es una prueba de sangre patentada (Siemens Healthineers, Erlagen, Alemania) que mide tres moléculas implicadas en el metabolismo de la matriz hepática (TIMP-1, PIIINP y AH), proporcionando un *score* que refleja la gravedad de la fibrosis hepática. Desde su aparición como el ELF original (OELF), el algoritmo se ha visto sometido a diversas modificaciones, como la eliminación del parámetro de la edad, habiéndose establecido diferentes puntos de corte [162, 163]. ELF ha mostrado una buena precisión en la predicción de la fibrosis hepática [77, 164], habiendo sido validado en diferentes patologías hepáticas crónicas como ALD [165], MASLD [166], cirrosis biliar primaria [167] e infección de hepatitis vírica [78, 168, 169]. Esta prueba es capaz de distinguir entre fibrosis grave, moderada y ausencia de fibrosis, con un AUC de 0,90, 0,82, y 0,76 respectivamente [170] (Tabla 2). Además, al igual que APRI y FIB-4, existe evidencia de la utilidad de ELF en la identificación temprana de pacientes con riesgo elevado de recurrencia de hepatitis C tras el trasplante hepático [171]. En pacientes con patología hepática crónica, existe una posible relación entre niveles elevados de ELF y peores resultados clínicos, lo que sugiere un valor pronóstico [76]. La guía de práctica clínica para la evaluación clínica y manejo de la MASLD de la AASLD recomienda su uso como prueba específica de segunda línea, siendo comparable a FibroScan en la evaluación de la fibrosis hepática [1, 172]. Sin embargo, en la interpretación de los resultados, y con el fin de minimizar la variabilidad de la prueba, hay que tener en cuenta diversos factores de influencia, como el sexo, la edad y el momento en que se realiza la prueba sanguínea [173].

#### Hepascore

Hepascore fue inicialmente validado para predecir los diferentes grados de fibrosis en pacientes con VHC. Esta prueba combina variables sociodemográficas como la edad y el sexo, con parámetros sanguíneos, entre los que se incluyen la bilirrubina, la gamma-glutamil transferasa, el AH y la α2-macroglobulina [80] (Tabla 1). Actualmente, es un algoritmo ampliamente utilizado para la detección de fibrosis moderada en multitud de patologías hepáticas crónicas [79, 174, 175]. Huang y col. [174] demostraron que Hepascore tiene mayor valor diagnóstico en la fibrosis moderada y avanzada en pacientes con CHC, CHB y ALD, que en aquellos con MASLD y coinfección de VIH y hepatitis vírica. Así mismo, se ha comparado con otros *score*s, mostrando valores diagnósticos considerablemente mayores en los pacientes con ALD que APRI y FIB-4, aunque similares a los de FibroTest y Fibrometer [85]. Por otro lado, en un grupo aleatorizado de pacientes alcohólicos, Chrostek y col. [176] concluyeron que Hepascore posee un valor diagnóstico inferior al de APRI, Forns y FIB-4, al emplear Fibrotest como matriz para comparar sus valores diagnósticos. Sin embargo, en pacientes con MASLD, Hepascor*e* es capaz de identificar la fibrosis hepática avanzada [177]. Hepascore también ha demostrado capacidad para predecir con exactitud los riesgos a largo plazo de eventos como descompensación y hepatocarcinoma, siendo ambos causa de mortalidad relacionada con enfermedad hepática en pacientes con disfunción metabólica asociada a MASLD [178]. Sin embargo, se observan una variación interindividual biológica en los niveles de AH en sangre extraídasin ayunas, tanto en sujetos sanos como en pacientes con patologías hepáticas, por lo que el sistema Hepa*score* se debería emplear con cautela en las determinaciones puntuales o en el seguimiento clínico [179].

#### FibroMeters

FibroMeters es una familia de paneles patentados formados por biomarcadores sanguíneos con diversas características, cuya flexibilidad permite adaptarlos a la causa de la enfermedad hepática crónica [180]. Estos incluyen biomarcadores directos como AH, α2-macroglobulina y biomarcadores indirectos como tiempo de protrombina, plaquetas, AST, ALT, GGT, bilirrubina, urea, ferritina, y otros datos como edad, peso corporal y sexo (Tabla 1). Los paneles de FibroMeters se desarrollaron y validaron inicialmente para establecer el grado de fibrosis en pacientes con CHB o hepatitis C crónica (CHC) [181] y MASLD [182]. Según Calès y col. [180], la prueba FibroMeter para la estadificación de la fibrosis en el CHC mostró un AUROC significativamente mayor que Fibrotest, Hepascore, APRI y FIB-4. En un metaanálisis en pacientes con CHC, FibroMeter demostró su superioridad con respecto a FibroTest y Hepascore en términos de valor diagnóstico global [183]. El FibroMeter estándar se amplió para mejorar su valor diagnóstico en la cirrosis, empleando coeficientes específicos con los mismos parámetros clínicos y sanguíneos que el FibroMeter estándar, resultando en un VPP del 100 % en los pacientes con VHC [184]. Además, en pacientes con MASLD, FibroMeter ha demostrado mayor precisión en el diagnóstico de la fibrosis moderada que APRI o NFS [185]. Los FibroMeter de segunda y tercera generación (V2G y V3G, respectivamente) fueron originariamente desarrollados para el diagnóstico de fibrosis moderada en pacientes con hepatitis C [186], aunque en los últimos años han sido validados también en pacientes con MASLD [182, 187]. La última versión es la elastografía de transición controlada mediante vibración (VCTE), que es una combinación de FibroMeter V3G y TE [188]. Es la prueba con la mayor precisión diagnóstica para la detección de fibrosis avanzada, frente a Fibrometer VG2 y Fibrometer MASLD [189], superando también a NFS y TE en el diagnóstico de fibrosis hepática grave en pacientes con MASLD [190]. Sin embargo, Guillaume y col. [187] obtuvieron la misma precisión para ELF y FibroMeterV2G en pacientes con MASLD. Además, FibroMeter VCTE posee una buena precisión diagnóstica, similar a la de TE, en la predicción de fibrosis grave en pacientes con hepatitis autoinmune, siendo incluso mejor en la colangitis biliar primaria, tal como observaron Zachou y col [191].

## Conclusiones

El diagnóstico temprano de fibrosis hepática en sus fases iniciales es esencial para garantizar una intervención clínica precisa y prevenir la progresión de fibrosis hepática a cirrosis y carcinoma hepatocelular. Así mismo, es importante diagnosticarla lo antes posible, ya que la fibrosis hepática está asociada a una mayor mortalidad global a largo plazo, necesidad de trasplante hepático y eventos hepáticos [192]. Los biomarcadores séricos se postulan como una excelente opción, ya que permiten un seguimiento continuado y son menos invasivos que la biopsia hepática. Todas estas escalas no invasivas, incluyendo los biomarcadores séricos directos e indirectos, poseen mayor sensibilidad, pero peor especificidad, por lo que deberían ser utilizadas para descartar a los pacientes con MASLD sin fibrosis avanzada [81], evitando así la realización de biopsias hepáticas innecesarias. Por sí solas, estas escalas no resultan válidas para establecer un diagnóstico preciso. El empleo de estos biomarcadores séricos e índices indirectos como primer paso, o la combinación de biomarcadores séricos con índices de fibrosis específicos, como ELF, o combinados con FibroScan o TE, son estrategias fiables recomendadas en las guías de práctica clínica para la estadificación de la fibrosis hepática en pacientes con diferentes patologías [16], siendo además un proceso diagnóstico económico. Además, algunos de estos biomarcadores, como FIB-4 o NFS son herramientas sencillas y económicas cuya implementación podría influir considerablemente en la identificación de pacientes con fibrosis hepática en sus fases iniciales, momento en el cual se puede revertir dicha patología [193, 194]. Aunque la totalidad de estos biomarcadores no invasivos e índices han sido estudiados en población de riesgo, podrían utilizarse en el cribado de grupos de pacientes de atención primaria, como pacientes con diabetes mellitus tipo 2, patologías relacionadas con el abuso del alcohol, factores de riesgo metabólico, o elevación de enzimas hepáticas, ya que sigue existiendo una amplia prevalencia de las enfermedades hepáticas crónicas entre la población general [195]. Por esta razón, la American Diabetes Association (ADA) y la European Associations for the Study of Diabetes (EASD), of the Liver (EASL) and of Obesity (EASO) recomiendan realizar un cribado de fibrosis hepática en todos los pacientes con diabetes mellitus tipo 2 [196, 197]. De este modo, todos los laboratorios deberían evaluar y definir la mejor estrategia diagnóstica, en consenso con los clínicos, con el fin de alcanzar el mejor rendimiento diagnóstico en su población diana.
